# Severe rhabdomyolysis and acute renal failure secondary to the use of simvastatin in undiagnosed hypothyroidism

**DOI:** 10.4103/0971-4065.41287

**Published:** 2008-01

**Authors:** F. A. Qari

**Affiliations:** Department of Nephrology, King Abdulaziz University, P. O. Box 80215, Jeddah 21589, Saudi Arabia

**Keywords:** Creatine kinase, hypothyroidism, rhabdomyolysis, simvastatin, acute renal failure

## Abstract

A 52-year-old Indian woman with underlying diabetes mellitus, hyperlipidemia and undiagnosed hypothyroidism presented with generalized musculoskeletal pain and oliguria for three days. The patient was taking 80 mg of simvastatin (initiated 20 days before) after cardiac catheterization for an inferior myocardial infarction. Laboratory evaluation revealed the following serum levels: creatine kinase, 81,660 U/L; aspartate aminotransferase, 2,497 U/L; alanine aminotransferase, 1,304 U/L; blood urea nitrogen, 88 mg/dL; creatinine, 5.1 mg/dL; free thyroxine (FT_4_), 12.6 Pmol/L and thyroid stimulating hormone, 22.7 uIU/L. Simvastatin was discontinued and the patient was administered forced alkaline diuresis. Her hypothyroidism was treated with thyroxine, which was continued after discharge. Her renal function recovered within two months. This case report discusses the higher incidence of rhabdomyolysis in patients with undiagnosed hypothyroidism receiving large doses of simvastatin.

## Introduction

Rhabdomyolysis is a well-known side effect of statin therapy. Manifestations are usually limited to muscle aches and weakness. When statins are used in patient with other risk factors for muscle injury, severe muscle damage may result. We report a case where introduction of simvastatin in a patient with unsuspected hypothyroidism caused severe rhabdomyolysis and acute kidney injury.

## Case Report

A 52-year-old Indian woman presented at King Abdulaziz University Hospital's emergency department with complaints of generalized, dull and constant myalgia. She expressed an 'inability to put her weight on her feet'. These symptoms persisted for three days and were associated with weakness, fatigue and a decreased urine output for two days. Her medical history included diabetes mellitus under insulin administration, hypertension, anemia secondary to fibroids and coronary artery disease with an inferior myocardial infraction (20 days before). The patient had no history of renal disease with a baseline creatinine of 106 μmol/L. Simvastatin (80 mg daily) was initiated by her cardiologist after she underwent cardiac catheterization 20 days prior to presentation. In addition, she was taking insulin, diuretics, losartan, aspirin and clopidogrel.

The patient's vital signs were as follows: temperature, 37°C; pulse, 60 beats per minute (paced); blood pressure, 130/65 mmHg and respiration, 18 breaths per minute. Physical examination showed paleness, obesity and dull appearance. Her system examination was largely unremarkable. No palpable goiter was observed on neck examination and there was no delayed relaxation of ankle jerk. Laboratory evaluation at admission revealed creatine kinase (CK) of 81,660 U/L; aspartate aminotransferase (AST), 2,497 U/L; alanine aminotransferase (ALT), 1,304 U/L; blood urea nitrogen (BUN), 88 mg/dL; creatinine, 5.1 mg/dL. Arterial blood gases demonstrated mild metabolic acidosis. Her serum potassium was 6.2 mmol/L and thyroid stimulating hormone (TSH), 22.7 U/L (normal range, 0.27-4.2) with a serum free thyroxine (FT_4_) of 12.6 Pmol/L. Her serum cholesterol level was 258 mg/dL; triglyceride, 196 mg/dL and low density lipoprotein (LDL), 134 mg/dL.

She was diagnosed with rhabdomyolysis and oliguric acute renal failure. Simvastatin was discontinued and she was administered forced alkaline diuresis (intravenous normal saline with bicarbonate). L-thyroxine (25 mcg) was administered initially and subsequently the dose was increased to 50 mcg daily. At discharge (15 days after hospitalization), laboratory results showed CK level of 1865 U/L and creatinine level of 1.8 mg/dL. Thyroxine administration was continued as an outpatient; however, no hydroxymethylglutaryl coenzyme (HMGCoA) reductase inhibitor was restarted in view of severe rhabdomyolysis. After six weeks, her CK was 151 U/L; creatinine level decreased to 1.4 mg/dL; AST level decreased to 186 U/L; and ALT decreased to 51 U/L. FT_4_ was 14.5 Pmol/L with a TSH of 2.06 U/L. On follow-up visit, she continued to report some mild residual weakness one month after hospitalization.

## Discussion

HMGCoA reductase inhibitors are effective in primary as well as secondary prevention of coronary disease.^1^ Therefore, these agents are used as the first choice for hypercholesterolemia in this subset of the population, especially atrovastatin and simvastatin.^2^ According to the research firm IMS Health, HMGCoA reductase inhibitors represent the highest class of drugs in the United States with regard to their sale; atorvastatin and simvastatin were the first and second most prescribed statins in 2006, respectively.^3^

They are usually well tolerated. However, they can cause a variety of musculoskeletal complications, including clinical mysotis and rhabdomyolysis.^4^ The reported risk of rhabdomyolysis with simvastatin monotherapy is low and dose related (0.02% at 20 mg daily, 0.07% at 40 mg daily and 0.3% at 80 mg daily).^5^ Several factors that increase the risk for both myopathy and rhabdomyolysis have been identified, including advanced age, chronic renal insufficiency, metabolic disorders such as diabetes or hypothyroidism, major surgery, alcohol abuse and medications that inhibit cytochrome P450 (CYP) 3A-4.^6^ Hypothyroidism frequently leads to asymptomatic mild to moderate elevation in the CK level.^7^ Marked elevation in CK with rhabdomyolysis has been reported only in a very small number of cases with unnoticed hypothyroidism using HMGCoA reductase inhibitors.^8^,^9^ Our patient received 80 mg of simvastatin without being diagnosed for primary hypothyroidism. The severity of hypothyroidism might be associated with an elevation in CK. The mechanism by which CK elevation occurs in hypothyroidism patients who are also receiving HMGCoA reductase inhibitors remains unclear.^10^ This group of drugs may represent an additional risk factor for myopathy and rhabdomyolysis in patients with hypothyroidism.

## Conclusion

We describe a case of severe rhabdomyolysis with undiagnosed hypothyroidism receiving a large dose of simvastatin. We emphasize the need to assess the possibility of hypothyroidism not only before treatment with HMGCoA reductase inhibitors but also during treatment, particularly marked increase in CK levels. Furthermore, in order to avoid the deleterious effects of these drugs, we recommend vigilant screening with regard to CK levels both upon initiation and during continued treatment with HMGCoA reductase inhibitors in individuals with hypothyroidism.
